# Dietary Iron Intake Impacts the Microbial Composition of the Murine Intestinal and Lung Microbiome

**DOI:** 10.3390/nu17162696

**Published:** 2025-08-20

**Authors:** Ama-Tawiah Essilfie, Alison Smith, Rebecca Watts, Pramila Maniam, Iain L. Lamont, David M. Frazer, Gregory J. Anderson, David W. Reid

**Affiliations:** 1 QIMR Berghofer Medical Research Institute, Brisbane, QLD 4006, Australia; ama-tawiah.essilfie@qimrb.edu.au (A.-T.E.);; 2 The School of Medicine, Herston Campus, The University of Queensland, Brisbane, QLD 4006, Australia; 3 The Department of Biochemistry, University of Otago, Dunedin 9012, New Zealand; 4 ^4^ The School of Biomedical Sciences, The University of Queensland, St Lucia, Brisbane, QLD 4067, Australia; 5 The School of Biomedical Sciences, Queensland University of Technology, Gardens Point, Brisbane, QLD 4000, Australia; 6 The Prince Charles Hospital, Brisbane, QLD 4032, Australia

**Keywords:** dietary iron, microbiome, colon, duodenum, lung, mouse

## Abstract

Background: Iron is an essential nutrient for many bacterial pathogens and normal cellular function and homeostasis of their hosts. Studies suggest that iron deficiency or overload may contribute to the pathogenesis of several chronic conditions and modify host–microbial interactions. In this study, we assessed the impact of varying dietary iron intakes on the microbiota of the intestinal tract and lungs of wild-type mice. Methods: Male C57BL/6J mice were fed either a standard pellet chow (high iron diet), a ferrous ammonium sulfate (FeAS)-supplemented diet or an iron-deficient diet for four weeks. Tissue from the lung, duodenum and colon was collected, and 16S rRNA gene fragments were pyrosequenced. Results: Total serum iron levels were negatively associated with richness of the lung microbiome (*p* = 0.035). In the murine lungs, there was no association between the iron diet and the overall lung microbiota community composition, but *Bacteroides* spp. were significantly enriched in the lungs of mice fed the FeAS diet (LDA score > 4, *p* < 0.05). The community composition of the intestinal microbiota changed significantly depending on the iron diet, with increased richness in the low-iron compared to the iron-supplemented groups (*p* = 0.053). In the duodenum, *Prevotella* spp. were reduced (Mean = 7.869, SEM = 3.464, *p* < 0.05), and *Desulfovibrio* species increased (Mean = 5.343, SEM = 1.362, *p* < 0.001) in iron-supplemented groups compared to the low-iron-diet group. In the colon, *Bifidobacterium* and *Bacteroides* species were reduced (Mean = 7.175, SEM = 2.246, *p* < 0.01 and Mean = 6.967, SEM = 1.834, *p* < 0.01 respectively), and *Pseudomonas* increased (Mean = 24.03, SEM = 8.919, *p* < 0.05) in mice on higher-iron diets compared to the low-iron diet. Discussion: This study demonstrates that dietary iron intake significantly impacts the intestinal microbiota and has a small, yet significant, effect on the lung microbiome in C57BL/6J mice. Whilst dietary iron content per se did not significantly modulate the composition of the lung microbiota, serum iron levels had subtle impacts on the community composition of the lung microbiota.

## 1. Introduction

Several human and animal studies have demonstrated that diet, specifically the intake of different proteins, fats and carbohydrates, can have a significant effect on intestinal microbiome composition [1]. Alterations in diet can change the gut microbial composition in as little as 24 h, but microbiome composition is also able to revert to baseline within 48 h of diet discontinuation [2].

Changes to dietary iron intake in human and animal studies can influence the population structure of bacterial communities that make up the gut microbiome, and they can be correlated with downstream impacts on immune regulation. This microbial dysbiosis is associated with diseases such as hypertension, Parkinson’s disease, asthma, type 2 diabetes, cancer and inflammatory bowel disease [3,4].

Iron is an essential biometal for the host in both health and disease, and also for almost all gut bacteria. As a result, several studies have investigated the effect of dietary iron on the gut microbiome, comparing the consequences of low versus high iron levels in the gut. Studies in East- and West-African children receiving either iron-supplemented biscuits or iron-fortified porridge showed increases in *Enterobacteria* and decreases in *Lactobacilli*, suggesting a shift towards a more pathogenic profile [5,6]. These children also showed signs of increased intestinal inflammation and a trend towards an increase in diarrhoea. Animal studies support these findings; iron-deprived mice and rats develop an increased abundance of *Lactobacillus* and a decrease in *Bacteroides* in the gut microbiota [7,8]. Recent studies have compared changes in the gut microbiome when iron-deficient animals received different iron supplements in the form of either conventional ferrous sulfate or Sucrosomial^®^ iron, which is a relatively new supplement [9]. The form of iron ingested significantly changed the microbial profile in the gut, with Sucrosomial^®^ iron increasing the abundance of potentially beneficial short-chain fatty acid–producing bacteria while decreasing more potentially pathogenic species.

The recognition of the inter-connectedness of the lung and gut microbiomes as part of the “gut–lung axis” suggests that diet could also potentially influence the lung microbiome and respiratory health outcomes [10,11,12]. Whether dietary iron impacts the lung microbiome is an important question, as there is strong evidence that lung iron homeostasis is abnormal in a number of chronic lung diseases, including the genetic disorder cystic fibrosis (CF), asthma, pulmonary fibrosis and chronic obstructive pulmonary disease (COPD) [13,14,15,16], and these diseases also have significant alterations in their microbiome. As a result, changes in diet which cause dysbiosis may influence pathology and clinical outcomes.

Although several studies have investigated the effect of either dietary iron or iron supplementation on gut microbiota [5,9,17,18,19,20,21,22,23,24,25,26], few have linked iron-related changes in the gut to changes in the lung microbiome [27,28,29]. The gut–lung axis (GLA) is proposed to work in a bi-directional manner, with the lung potentially being the source of some gut microbes and vice versa [30,31]. Apart from microbes, there are several factors that influence and regulate the GLA, including immune cells and their mediators, microbe-derived metabolites and microbe crosstalk between the lung and gut. Gut dysbiosis, caused by diet, antibiotics, infection or smoking, can cause an inflammatory response that damages the epithelial membrane barrier, allowing metabolites, commensal microbes and inflammatory cytokines to leak into the lung via systemic circulation [32]. This cross-communication is important in both health and disease, so we hypothesise that higher levels of dietary iron intake will adversely affect the gut microbiome and that these changes will induce a change in the lung microbiome via the gut–lung axis. Despite the large number of studies investigating the human gut microbiome, these are still far from exhaustive, and there are always many cofounders in disease settings, such as the impact of drug treatments. We have, therefore, chosen to use an animal model to investigate our hypothesis, as this is a well-established, proof-of-concept starting point. The precise effect of different dietary iron intake levels on the gut microbiome is a field of research still in its infancy, and although there are no obvious adverse effects from making small changes to iron intake on the human body as a whole, the changes to the microbiome of the gut and the downstream effects of those changes are largely unknown, but likely to be significant. The mouse model gives us a controlled environment within which this can be investigated. We, therefore, investigated how dietary iron intake in wild-type C57BL/6J mice would influence the composition of the microbiota of the gastrointestinal tract (GIT) and whether those changes would alter the lung microbiome.

## 2. Materials and Methods

### 2.1. Experimental Approach

**Scheme 1:**
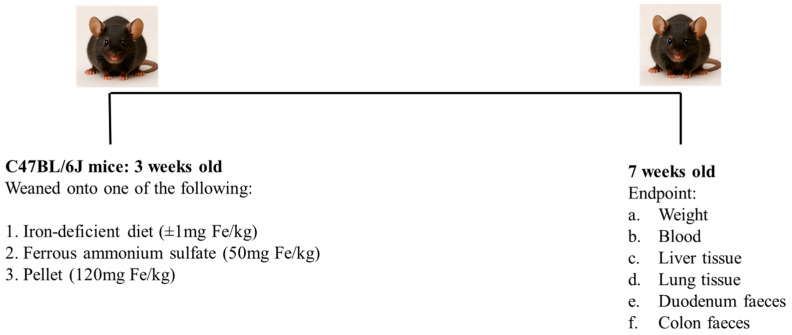
Timeline of experimental interventions. Upon weaning, 3-week-old mice were placed on different iron diets. At the endpoint, 4 weeks later, weight was measured and blood, liver and lung tissue and faeces collected for further 16S analysis.

Upon weaning, specific-pathogen-free, three-week-old C57BL/6J mice were randomly assigned into three groups of six mice and fed one of three diets. The mice were housed in groups of three mice/cage, with the same cage components, such as bedding material, on the same animal housing rack for the entirety of the study. Animals in group 1 were fed an iron-deficient diet ad libitum based on AIN93G (Specialty Feeds, Glen Forrest, Australia; about 1 mg/kg iron, n = 6); those in group 2 were fed a ferrous ammonium sulfate-supplemented diet ad libitum (AIN93G supplemented with FeAS; 50 mg iron/kg, n = 6); and those in group 3 were fed a standard mouse pellet chow ad libitum (Norco Stockfeed, Lismore, Australia), which is intrinsically high in iron content (120 mg iron/kg as ferric citrate, n = 6). After four weeks, the mice were weighed and euthanised, and faeces and tissue samples from the duodenum, colon and lung were collected, snap frozen and stored at −80 °C (Scheme 1 below). All mice were housed in the animal facility at the QIMR Berghofer Institute under identical conditions, and all processes and procedures were approved by The QIMR Berghofer Animal Ethics Committee.

### 2.2. Iron Concentration Measurements

The iron status of the mice at the end of the dietary intervention was assessed by measurement of their liver iron concentration and total serum iron levels [33,34]. Approximately 20 mg of dried liver was used to measure hepatic iron levels, as previously described by Frazer and colleagues [33]. Briefly, 1 mL of 3 M hydrochloric acid and 0.6 M trichloroacetic acid solution were added to each sample and to the relevant amounts of the iron stock to make up a standard curve (0–90 µg/mL). The samples and iron standards were incubated overnight at 65 °C, then 4 µL of the sample (standard or liver sample) was added to freshly prepared chromogen reagent. After a 30 min incubation at room temperature, the absorbance was measured at 535 nm with a plate reader, and, using the standard curve, the amount of iron was determined. Serum iron was measured using a colorimetric assay according to the manufacturer’s instructions (Pointe Scientific, Canton, OH, USA).

### 2.3. Microbiome Analysis

Genomic DNA was extracted from the tissue samples using the PureLink^®^ Genomic DNA extraction kit according to the manufacturer’s instructions (Invitrogen, Waltham, MA, USA). 16S rRNA was amplified using the universal primer set 939F (TTGACGGGGGCCCGCAC) and 1492R (TACCTTGTTACGACTT) that binds to the conserved region flanking the variable region V5-V9 and sequenced using bacterial tag-encoded FLX amplicon pyrosequencing, as previously described [35]. A sample comprising only the extraction kit buffer was co-sequenced to assess reagent contamination (signal was subtracted from experimental samples). The data were processed using the QIIME2 (v2020.11) software [36] and analysed using the Calypso platform [37].

### 2.4. Data Handling

The sample size was determined by the primary outcome measure 16S sequencing. All six mice/group were included in each experiment; however, the final results contained n = 4–6 data points. Data points were removed after an outlier test (GraphPad Prism, v9.3.1) revealed outliers in the data. The authors conducting the experiments were aware of the different group allocations while conducting the experiments and preparing the final results’ graphs and figures. Microbiome analysis was conducted in a blinded fashion by an external service provider.

### 2.5. Statistical Analysis

Iron indices and weight data were analysed using a one-way analysis of variance (ANOVA) with a Bonferroni post-test or Pearson correlation. The data are displayed as mean ± SEM.

The microbiome data were imported into Calypso using total sum normalisation combined with square root transformation. Differences in taxa between different diet groups were compared using ANOVA with a *p*-value correction using the false discovery rate (FDR). Pearson correlations were calculated to identify the relationships between the abundance of genera in the duodenum, colon and lung. The microbial diversity at each anatomical site, depending on the different iron diets, was assessed with Shannon diversity and richness and evaluated using a one-way ANOVA test.

## 3. Results

### 3.1. Iron Status and Weight

**Figure 1:**
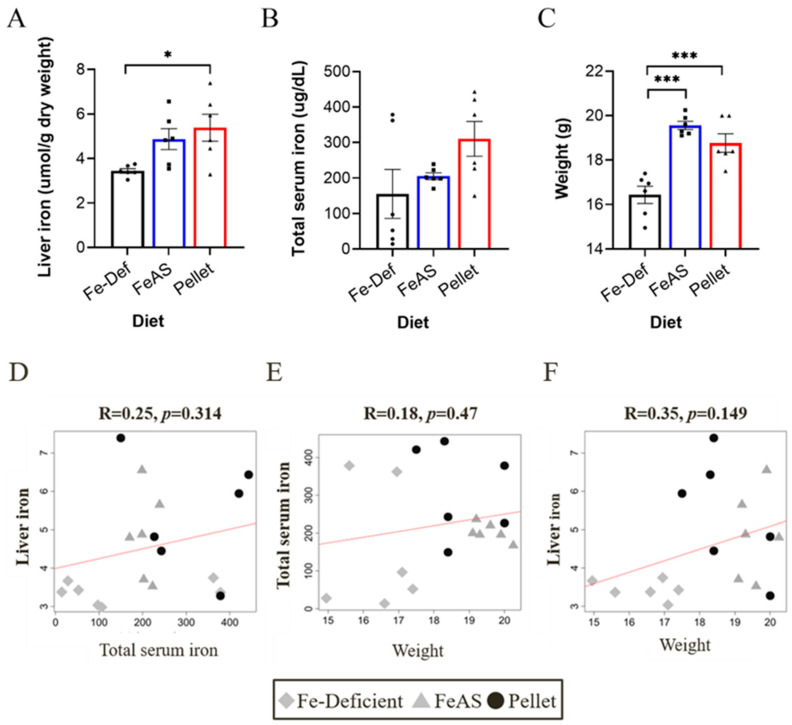
Iron indices and weight of mice fed different dietary iron intakes. (**A**) Liver iron ($\eta_{p}^{2}\;$= 0.60), (**B**) total serum iron ($\eta_{p}^{2}$ = 0.40) and (**C**) weight ($\eta_{p}^{2}\;$= 0.71) of mice fed an iron-deficient (Fe-Def), pellet (high-iron) or FeAS (ferrous ammonium sulfate)-supplemented diet. Relationship between liver iron and total serum iron (**D**), serum iron and weight (**E**) and liver iron and weight (**F**) were assessed. Pairwise group comparisons were performed using t-tests and are annotated using asterisks (*p*-value: * < 0.05, *** < 0.001). Correlation between liver iron, total serum iron and weight was calculated using Pearson correlation (**D**–**F**); iron concentration (µM/g dry weight), total serum iron (µg/dL) and weight (g). For each group in every analysis, n = 6. $\eta_{p}^{2}$ = effect size (partial eta squared).

Three-week-old mice were weaned onto either an iron-deficient diet (Fe-Def), an FeAS-containing diet (50 mg/kg iron), or a relatively high iron standard pellet diet (120 mg/kg) and maintained on these diets for four weeks before samples were collected. Liver iron levels were significantly increased in mice fed the pellet diet compared to those fed the iron-deficient diet (Figure 1A), while total serum iron levels showed no significant differences but trended towards an increase in the pellet diet (*p* = 0.09; Figure 1B). Mice on the iron-deficient diet had significantly lower weights than the mice on the FeAS and pellet diets (Figure 1C). This trend may be attributed to the lower amount of iron in the diet of these mice, though there is also a slight difference in the make-up of the iron-deficient diet compared to the FeAS diet, so the variation in weight may also be affected by a variation in protein and carbohydrates. There was no correlation between mouse weight, liver iron content and serum iron levels (Figure 1D–F).

### 3.2. Iron Supplementation Has a Small Effect on the Microbiome of the Lung

**Figure 2:**
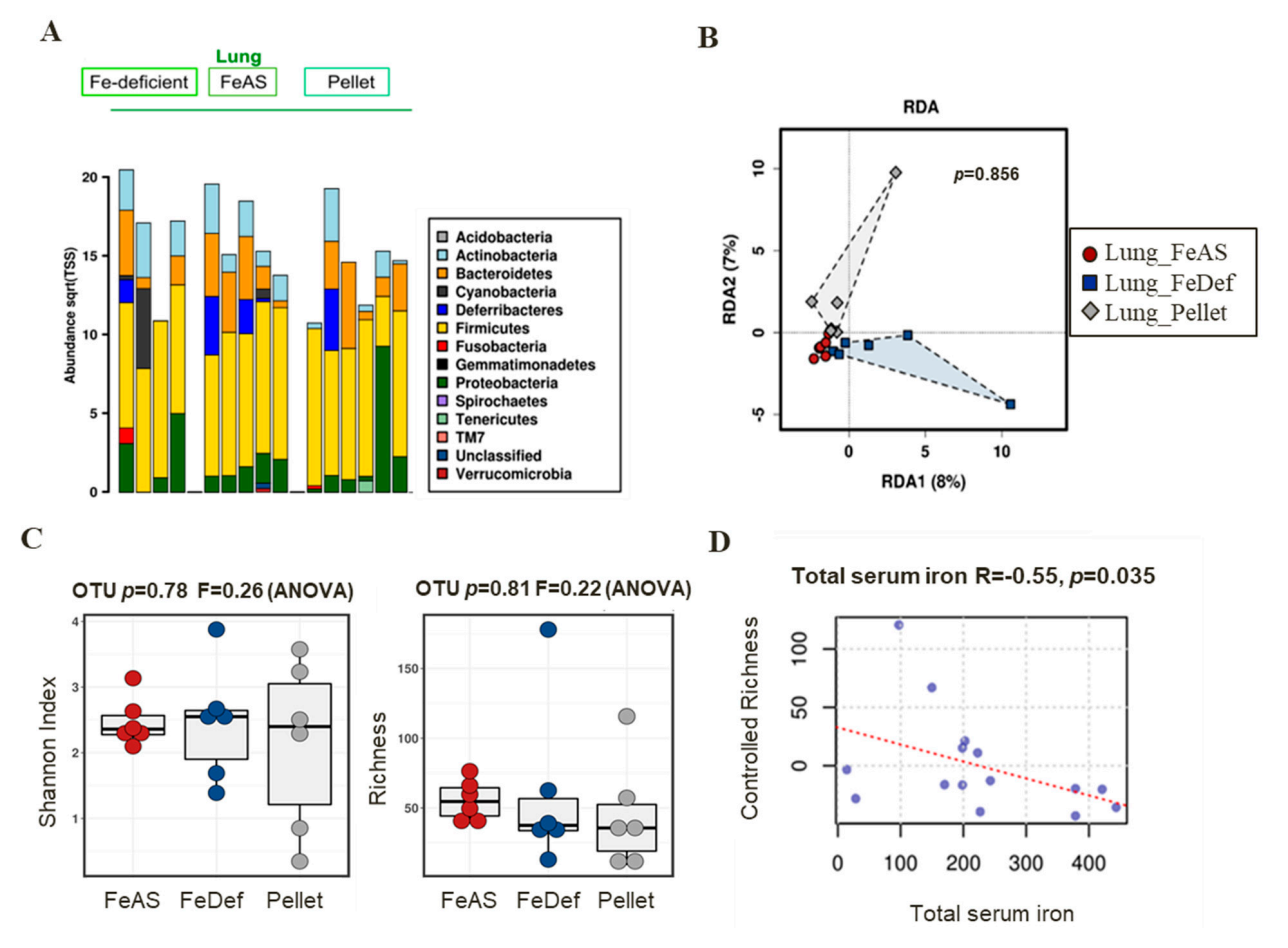
Taxonomic profile of the microbiome in the lung. (**A**) Bar chart of the microbiome composition at the phyla level in the lung after 4 weeks of different iron diets, (**B**) redundancy analysis (RDA) plot, (**C**) Shannon index and richness plots and (**D**) richness correlation with total serum iron after correcting for weight. Fe-deficient diet (FeDef, 0 mg/kg iron), ferrous ammonium sulfate diet (FeAS, 50 mg/kg iron) and pellet diet (120 mg/kg iron) and OTUs (operational taxonomic units). N = 4–6 for the phyla-level microbiome composition data, and n = 6 for all other groups in the remaining analyses.

At the phylum level, the mouse lung microbiome was dominated by *Firmicutes* and *Bacteroidetes*, which has been shown by others, and this remained largely unchanged after feeding mice diets of differing iron contents (Figure 2A). A redundancy analysis (RDA) plot demonstrated that while the different diets led to some clustering, this did not change significantly when the iron content of the diet was altered (Figure 2B). There was no difference in microbial diversity (Shannon index) or richness in the lung when diets of differing iron content were used (Figure 2C). Interestingly, total serum iron showed a significant negative correlation with richness (Figure 2D). Furthermore, a comparison of the lung microbiome in the mice fed the three different diets using linear discriminant analysis (LDA) effect size revealed an association between operational taxonomic units (OTUs) related to *Bacteroides* spp. and the FeAS diet (LEfSe, LDA score > 4; Supplementary Figure S1A–C). The family *Bacteroidaceae*, the genus *Bacteroides* and OTUs related to *Bacteroides* spp. were enriched in the lung microbiome of the mice fed the FeAS-supplemented diet compared to the iron-deficient and normal pellet diets (Supplementary Figure S1D,E).

### 3.3. Iron Supplementation Significantly Changes the Microbiome of the Duodenum

**Figure 3:**
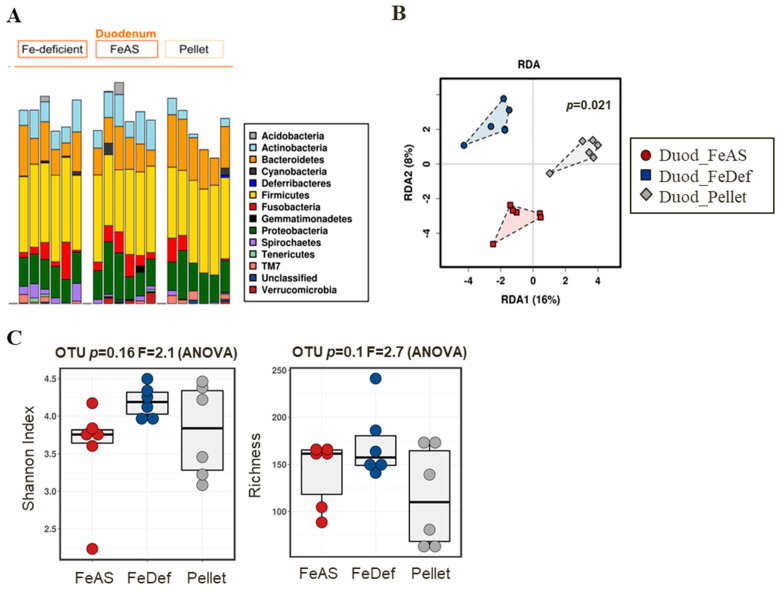
Microbiome profile in the duodenum. (**A**) Bar chart of the microbiome composition at the phyla level in the duodenum after 4 weeks of different iron diets, (**B**) redundancy analysis (RDA) plot and (**C**) Shannon index and richness plots for the duodenum. Fe-deficient diet (FeDef, 0 mg/kg iron), ferrous ammonium sulfate diet (FeAS, 50 mg/kg iron) and pellet diet (120 mg/kg iron), OTUs (operational taxonomic units). There was an n = 6 for each group in every analysis.

As in the lung, and as expected, the dominant bacterial phyla in the duodenum were *Firmicutes* and *Bacteroidetes*. In addition, *Proteobacteria* and *Actinobacteria* were increased in abundance in the duodenum compared to the lung. The iron-deficient and FeAS diets had a similar abundance of *Proteobacteria* and *Actinobacteria*; however, the pellet diet seemed to reduce the abundance of *Actinobacteria* (Figure 3A). Redundancy analysis showed significant differences between all diets (Figure 3B), while microbial diversity and richness were similar across the different diets (Figure 3C). Richness tended to be decreased in the pellet-fed mice group compared to the iron-deficient mice group (*p* = 0.057).

### 3.4. Iron Supplementation Significantly Changes the Microbiome of the Colon

**Figure 4:**
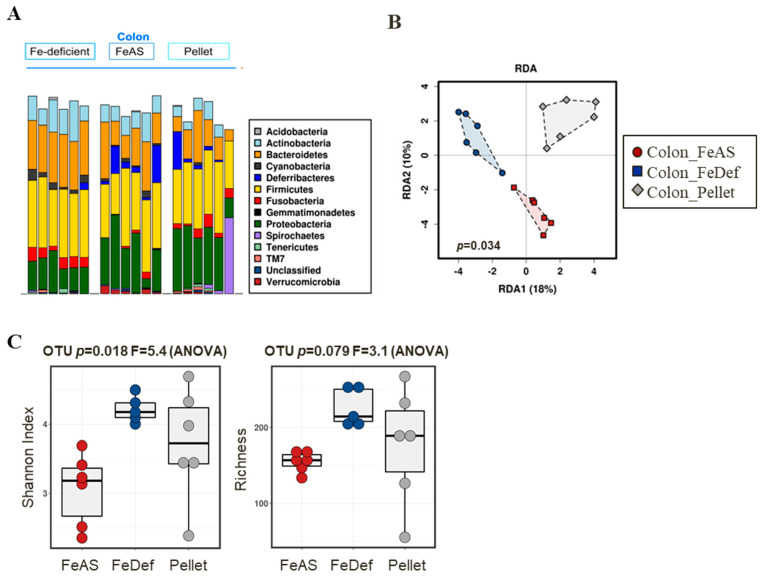
Microbiome profile in the colon. (**A**) Bar chart of the microbiome composition at the phyla level in the colon after 4 weeks of different iron diets, (**B**) redundancy analysis (RDA) plot and (**C**) Shannon index and richness plots for the colon. Fe-deficient diet (FeDef, 0 mg/kg iron), ferrous ammonium sulfate diet (FeAS, 50 mg/kg iron) and pellet diet (120 mg/kg iron), OTUs (operational taxonomic units). N = 5–6 for microbiome diversity data, and n = 6 for all other groups in the remaining analyses.

In the colon, *Firmicutes*, *Proteobacteria, Bacteroidetes* and *Actinobacteria* were again the main bacterial phyla. *Proteobacteria* were increased and *Bacteroidetes* decreased in animals fed the higher-iron diets compared to those fed the FeDef diet (Figure 4A). Similar to the duodenum, redundancy analysis demonstrated distinct clustering and a significant difference between all three diets (Figure 4B). However, unlike the lung and duodenum, the diet significantly altered the microbial diversity and richness in the colon. Interestingly, the iron-deficient diet had the highest microbial diversity index and richness score, and the FeAS diet had the lowest diversity and richness score (Figure 4C).

### 3.5. Changes in Microbial Taxa Between Diets in the Lung, Duodenum and Colon

**Figure 5:**
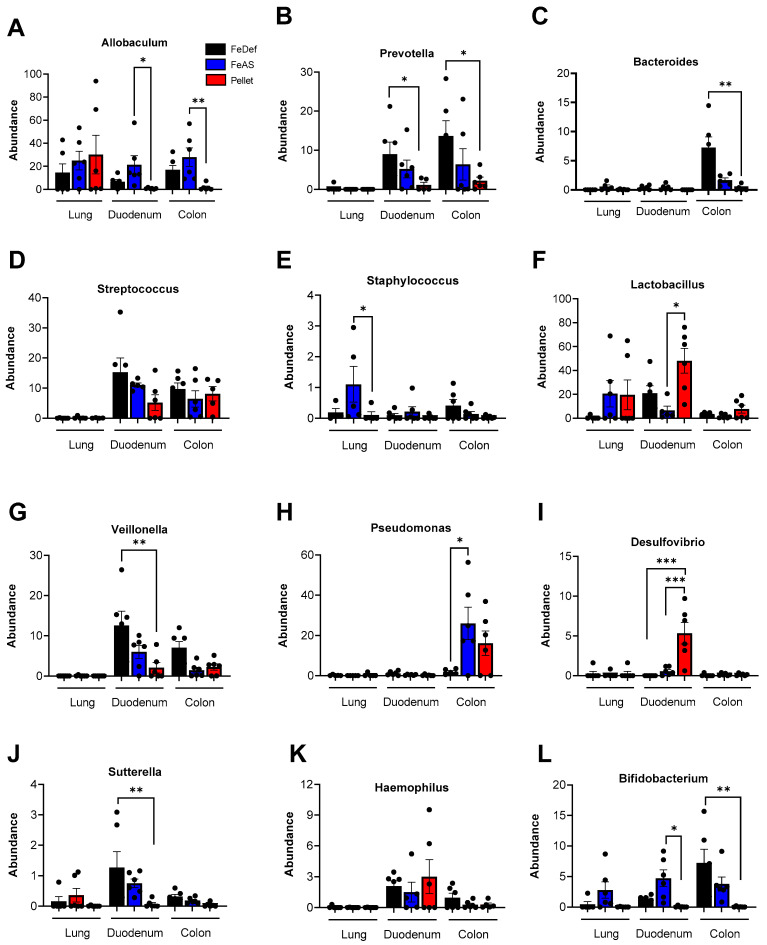
Changes in bacterial genera in lung, duodenum and colon. Mice fed either iron-deficient, FeAS or pellet diet were analysed for microbial genera in lung, duodenum and colon. Relative abundance of (**A**) *Allobaculum* ${(\eta }_{p}^{2}$ = 0.26), (**B**) *Prevotella* ${(\eta }_{p}^{2}$ = 0.76), (**C**) *Bacteroides* ${(\eta }_{p}^{2}$ = 0.71), (**D**) *Streptococcus* ${(\eta }_{p}^{2}$ = 0.48), (**E**) *Staphylococcus* ${(\eta }_{p}^{2}$ = 0.33), (**F**) *Lactobacillus* ${(\eta }_{p}^{2}$ = 0.44), (**G**) *Veillonella* ${(\eta }_{p}^{2}$ = 0.59), (**H**) *Pseudomonas* ${(\eta }_{p}^{2}$ = 0.58), (**I**) *Desulfovibrio* ${(\eta }_{p}^{2}$ = 0.70), (**J**) *Sutterella* ${(\eta }_{p}^{2}$ = 0.42), (**K**) *Haemophilus* ${(\eta }_{p}^{2}$ = 0.40) and (**L**) *Bifidobacterium* ${(\eta }_{p}^{2}$ = 0.50) were quantified. N = 6 for each group in every analysis. $\eta_{p}^{2}=$ effect size (partial eta squared), *p*-value * < 0.05, ** < 0.01, *** < 0.001.

To narrow down the classification of microbes, several bacterial genera were assessed after the mice were fed a low-iron diet (FeDef), a medium-iron diet (FeAS) or a pellet diet. A small number of bacteria were changed in the lungs as a result of these diets; however, there were several significant changes in the gut (duodenum and colon). *Allobaculum* was significantly reduced in mice fed the pellet diet in both the duodenum and colon (Figure 5A). We showed that in the gut, the levels of bacteria in the class *Bacteroidia*, *Prevotella* and *Bacteroides* were significantly reduced in the mice fed the pellet diet compared to the iron-deficient animals (Figure 5B,C). When we further examined the *Bacilli* class, we found a variety of responses. There were no changes in *Streptococcus* (Figure 5D), *Staphylococcus* was lower in the lungs of mice on the FeAS-supplemented diet relative to those on the pellet diet (Figure 5E), there was an increase in *Lactobacillus* in the duodenum of mice fed the pellet diet (Figure 5F) and, finally, there was a decrease in *Veillonella* in the gut of mice fed the pellet diet compared to the iron-deficient animals (Figure 5G). When we examined the *Gamma-proteobacteria* class of pellet-fed animals, we found increases in *Pseudomonas* in the colon (Figure 5H) and *Desulfovibrio* in the duodenum (Figure 5I) and decreases in *Sutterella* in the duodenum (Figure 5J) but no changes in *Haemophilus* (Figure 5K). Finally, we showed that *Bifidobacterium* was decreased in the gut of mice fed the pellet diet compared to those fed the FeAS or FeDef diets (Figure 5L).

## 4. Discussion

This study is one of the few to investigate the simultaneous effects of dietary iron intake on the microbiota of the lung, duodenum and colon in C57BL/6J mice. Our findings demonstrate that the community composition of the microbiota in the duodenum and colon changes significantly with dietary iron intake, which is consistent with previous studies [5,9,18,19,21,22,28]. The lung microbiota was not significantly affected by the different iron diets per se, but *Bacteroides* spp. showed a trend of increased abundance in the mice fed the FeAS-supplemented diet compared to the mice on a low-iron diet. Interestingly, as serum iron levels increased, microbial richness of the lung decreased, which suggests that systemic iron levels may potentially modulate the lung microbiome.

Reports on the most abundant bacteria in the murine lung microbiome are conflicting [38,39,40], which probably reflects different sample collection methods (bronchoalveolar lavage [32] vs. lung tissue), sequencing technology, PCR primer selection, mouse strain and the environment within which mice are housed, i.e., differing resident microbes in the pellet chow, cage bedding and drinking water. Sequencing of lung tissue in C57BL/6J mice has demonstrated a microbiome dominated by *Ralstonia*, *Lactobacillus*, *Enterobacteriaceae* and *Sphingomonas* species [38]. In another study, BAL and lung tissue samples demonstrated that the murine lung microbiome was mainly composed of the genera *Staphylococcus*, *Massilia*, *Corynebacterium*, *Pseudomonas* and *Streptococcus* [40]. A study by Scheiermann et al. categorised the murine lung microbiome in female Balb/cJ mice into three distinct pneumotypes [39]. The first pneumotype (a third of the lung samples) was considered sterile after correcting for contamination introduced by sequencing. A second pneumotype was characterised by a high abundance of the genera *Streptococcus*, *Haemophilus* and *Actinobacillus*, which overlapped with the microbiome of the murine oral cavity. The third pneumotype described was dominated by chow- and cage-bedding-associated bacteria. Thus, there is some variability in the murine lung microbiota depending on the study variables and environmental factors in experimental animals living under artificial conditions.

In our study, the genera *Allobaculum*, *Lactobacillus*, *Bifidobacterium* and *Streptococcus* were the most abundant taxa in the murine lung microbiome. *Lactobacillus* and *Streptococcus* have previously been reported in the murine lung microbiome [39], but to our knowledge, this is the first report of the presence of *Allobaculum* and *Bifidobacterium*. *Allobaculum* and *Bifidobacterium* are common members of the murine gut flora, and it is conceivable that their presence represents inhalation of faecal material by the mice from the environment in the cage. Future studies would need to investigate how bedding and faeces in the cage may contribute to the lung microbiome. The few studies that have investigated the role of *Allobaculum* in the gut demonstrated that *Allobaculum* induced an increase in T-helper 17 cells, disrupted the intestinal barrier and promoted a pro-inflammatory milieu. Although studies have shown correlations between *Allobaculum* abundance in the gut and changes in the lung microbiome composition, there are no data demonstrating its role specifically in the lung [41,42,43].

*Bacteroides* spp., which differed in relative abundance between low and high iron diets in the lung in this study, are also normal commensals of the gut, and the presence of *Bacteroides* spp. has been reported in other studies of the murine lung [38]. *Bacteroides* spp. have also been identified in BAL samples from human studies [44]. In our study, *Bacteroides* spp. were enriched in the lungs of animals fed the FeAS diet but not the iron-deficient or pellet mice dietary groups. *Bacteroides* spp. were also present in low abundance in the pellet-fed mice but were highly abundant in the colons of the mice receiving both the FeAS-supplemented and iron-deficient diets.

Within each dietary iron group, the lung microbiome was more heterogeneous between individual mice than in the duodenum or colon. This individual heterogeneity of bacterial communities in the lung microbiota is consistent with previous reports [39]. Interestingly, serum iron levels were negatively associated with overall diversity of the lung microbiome, whilst liver iron, which is generally considered a reliable measure of systemic iron stores, was not. A limitation of this lung microbiome proof-of-concept study is the small sample size and the variation of lung microbiota composition that we found within each diet group. However, although our study was not sufficiently powered to detect changes at an individual OTU level, the relationship between serum iron and lung microbiome diversity suggests that the lung microbiome is sensitive to systemic iron levels. As the lung microbiota represent a low-biomass sample, increasing the sample size will also decrease the within-group variation and strengthen the reliability of the data. We did not determine whether increased serum iron levels resulted in higher lung iron content, and the mechanism by which serum iron modulates the lung microbiome needs to be further investigated in a larger study. Our group and others have demonstrated an increase in iron levels and iron metabolism dysregulation in the airways and lungs of CF, asthma and COPD patients [13,14,16,45,46]. This dysfunction in iron homeostasis induces pro-fibrotic responses in fibroblasts and airway smooth muscle cells in asthma and fibrotic lung disease models [15,16], induces ferroptosis of airway epithelial cells in CF and COPD [47,48] and induces pro-inflammatory responses in all three airway diseases. These changes in iron are associated with the pathogenesis of severe disease and increased susceptibility to respiratory infections [16,49,50,51]. Interestingly, in humans, iron infusion resulted in a rapid rise in sputum iron levels in adult CF patients, suggesting that iron may transit from the vascular compartment into the airway lumen relatively easily, particularly in the setting of the mucosal inflammation that is present in CF [52]. The microbiome is also significantly altered in all three of these diseases, and this is also thought to play a critical role in disease pathogenesis [53,54,55,56,57,58,59,60,61,62,63]. Our findings in mice may, therefore, have implications for chronic airway diseases where abnormal lung iron homeostasis plays a pivotal role, and in these diseases, an abnormal lung microbiome likely contributes to airway inflammation and disease progression; in this context, iron moving from the circulation into the airway lumen may be an important modifying factor.

In contrast to the lung, the gut is very densely populated with diverse bacterial communities; in fact, the gut contains the largest microbiome in the human body [64]. Within the gut, the human host and resident microbiota compete for iron acquisition, and the microbiota are able to regulate host functions to increase access to iron. Dietary iron is, therefore, a strong regulator of the community composition and behaviour of the gut microbiome [65]. The phyla *Firmicutes* and *Bacteroidetes* dominate both the mouse and human gut from early life, with changes in the ratio between these two phyla occurring at different time points of life [66]. Our findings are consistent with these observations, and, interestingly, a low-iron diet in our study was associated with an increase in the relative abundance of both *Bacteroidetes* and *Firmicutes* in seven-week-old mice. This may be due to the fact that for many of these species, the heme requirement for survival is low due to their being able to produce energy by non-respiratory fermentative methods that do not rely on heme [67]. *Bifidobacterium* is also increased in the colon of mice fed a low-iron diet compared to those fed a pellet diet. There are several studies showing the health benefits of *Bifidobacterium*, with a high relative abundance in the gut reported during early infancy [68,69,70,71]. These early-life bacterial communities have been shown to decrease in abundance with age, and decreased *Bifidobacterium* is associated with asthma [72] and irritable bowel syndrome [73], and it may potentially contribute to dysbiosis and lung disease in CF [74]. Further research is needed to determine how constituents of the gut microbiota such as *Bifidobacterium* may interact with other species, such as *Prevotella* and *Bacteroides*, to influence disease pathogenesis and progression.

In the duodenal microbiota, iron supplementation in our study increased the relative abundance of the sulfate-reducing bacterium *Desulfovibrio*, consistent with a mouse model of Crohn’s disease in which a low-iron diet was associated with a decreased *Desulfovibrio* abundance [75]. *Desulfovibrio* spp. are predominantly sulfate-reducing bacteria that produce the metabolite hydrogen sulfide, which has far-reaching effects on host health. *Desulfovibrio* spp. are one of the 20 most abundant genera in humans and mice; they are generally considered to be harmful in large amounts and are associated with a number of chronic diseases, such as ulcerative colitis, obesity and Parkinson’s disease. However, they are also thought to provide some benefits to the host [76]. A handful of studies have shown in animal models that an increase in iron intake results in an increase in *Desulfovibrio;* however, it remains unclear why this occurs, and we cannot answer this question from our data [77,78]. Taken together with our results, these studies do, however, suggest that an increase in iron, irrespective of the source, may directly induce changes in the lung microbiome that contribute to disease pathogenesis but that also have several effects in distal sites due to the release of metabolites such as hydrogen sulfide. In contrast, we found that *Sutterella*, *Veillonella dispar* and *Prevotella melaninogenica* were increased in the duodenum in the low-iron-diet group. *Veillonella dispar* is normally isolated from the oral cavity and small intestine of humans [79], and it is known to possess a genome adapted to scavenging iron from its environment, while *P. melaninogenica* also has the capacity to scavenge iron through heme and hemoglobin uptake mechanisms. This allows it to survive in low-iron environments where only organic iron sources may be available [80].

Our observations that dietary iron changed the composition of the duodenal and colonic microbiota can be considered in light of the growing body of evidence that suggests the gut microbiome plays a role in the maintenance of human health [81,82,83,84]. The impact of dietary iron intake on the composition and behaviour of the gut microbiota may, therefore, have far-reaching effects on overall health. This needs further investigation, particularly given the widespread and sometimes indiscriminate use of iron supplements in developed countries and the high prevalence of iron deficiency as a component of malnutrition in many parts of the world. The putative “gut–lung axis” concept is also gaining substantial traction, and mouse studies have shown that changes in the composition of the gut microbiome modulate lung T cell population and activation profiles and, thus, may impact the host immunity and lung disease phenotype [10]. Although we did not assess downstream effects on host immunity, our demonstration of a significant perturbation of the dynamics of the gut microbiota depending on dietary iron may well have systemic implications, such as changes in the exacerbation of systemic and/or lung inflammation [85,86]. There are a number of pathways that may be important in how dietary iron may influence distal microbiota. As discussed above, studies have shown that diet (and other factors) can adversely affect gut microbe communities. This gut dysbiosis can directly disrupt epithelial tight junctions by altering junctional complexes to increase intestinal permeability [87,88,89] and also increase systemic exposure to PAMPs and bacterial metabolites that can induce a pro-inflammatory response, including increased T-helper cell (Th) type-1 and Th17 cells [90,91]. Alternatively, metabolites could also be transported from the intestinal lumen into the lamina propria and be transported to the lungs via the systemic circulation. Together, these potential mechanisms could explain how changes in nutrient intake could affect the lung microbiome.

To validate and expand on our data, future studies should include investigating the metabolite changes that occur as a result of dietary intervention as well as subsequent changes to inflammatory markers and immune cells. To further this work, future studies should also quantify iron levels and iron modulatory proteins in the lungs and gut across different timepoints. Our data capture a snapshot of the microbial communities after four weeks on the different diets; however, there may well be more significant changes that arise after several months on these diets. These data would inform how iron-induced changes to the microbiome affect the critical underlying processes in chronic airway diseases and, potentially, other distal organ diseases.

## 5. Conclusions

This proof-of-concept study demonstrates that dietary iron modulates the community composition of the gut microbiome and that systemic iron levels may influence the diversity of the lung microbiome in C57BL/6J mice. These findings may be of particular relevance in human diseases where an altered lung microbiome already exists and lung iron homeostasis may be intrinsically abnormal. The potential for inappropriate dietary iron supplementation to have adverse effects on the gut and lung microbiome in diseases such as CF and COPD needs to be considered and explored in further detail.

## Data Availability

The original contributions presented in this study are included in the article/supplementary material. Further inquiries can be directed to the corresponding author.

## Supplementary Materials

The following supporting information can be downloaded at https://www.mdpi.com/article/10.3390/nu17162696/s1: Figure S1: Dietary iron intake changes the Bacteroides genus in the lung.
