# Supplementary material for: Dietary Iron Intake Impacts the Microbial Composition of the Murine Intestinal and Lung Microbiome
Source: Nutrients. 2025 Aug 20;17(16):2696. doi: 10.3390/nu17162696 (PMC12389207; doi:10.3390/nu17162696)
Supplement: Supplementary file 1 [file nutrients-17-02696-s001.zip › nutrients-3766264-supplementary.pdf]

Supplementary Figure S1

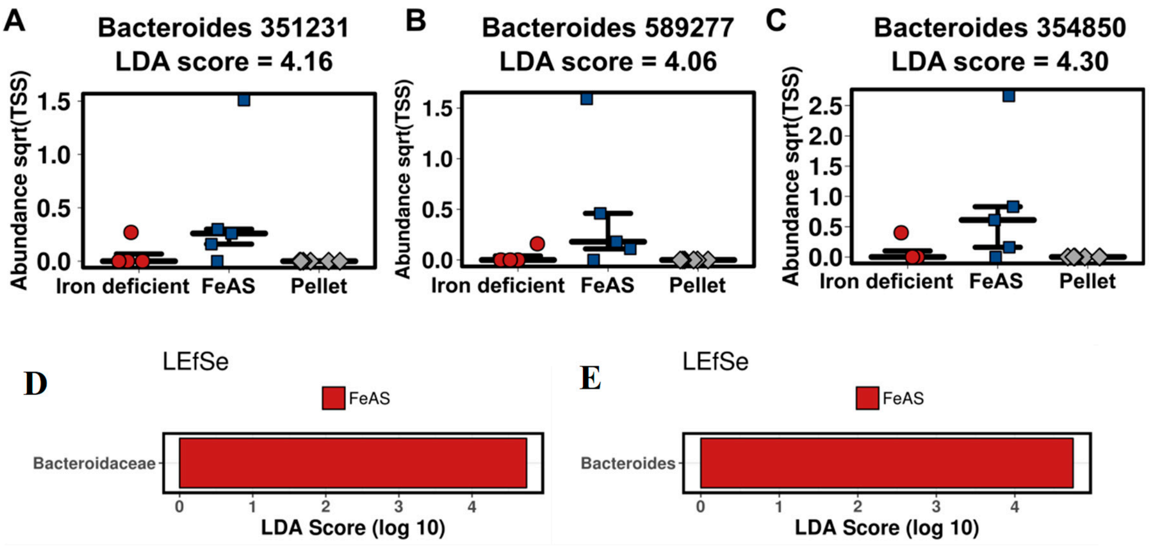

Supplementary Figure S1: Changes in taxa between diets differing in iron amount in the lung. (A-C) OTUs enriched in FeAS diet based on linear discriminant analysis scores (LDA) taking into account the effect size (LEfSe), (D) the family *Bacteroidaceae* and (E) the genus *Bacteroides* were enriched in the lung microbiome of mice fed FeAS-supplemented diet compared to the iron-deficient and pellet diets.
